# The effects of cold compress and transcutaneous electrical nerve stimulation on the pain associated with chest tube removal among patients with coronary bypass grafting

**DOI:** 10.1186/s13019-023-02182-9

**Published:** 2023-05-25

**Authors:** Fatemeh Hatefi, Majid Kazemi, Parvin Manglian, Dadullah Shahi Moridi, Shahin Heydari, Hadi Hasani

**Affiliations:** 1 Department of Medical Surgical Nursing, Faculty of Nursing and Midwifery, Rafsanjan University of Medical Sciences, Rafsanjan, Iran; 2 Department of Medical Surgical Nursing, Faculty of Nursing and Midwifery, Non-Communicable Disease Research Center, Rafsanjan University of Medical Sciences, Nurse Street, Rafsanjan, Iran; 3 Department of Critical Care Nursing, Faculty of Nursing and Midwifery, Kerman University of Medical Sciences, Kerman, Iran; 4 Department of Basic Science, Faculty of Medicine, Rafsanjan University of Medical Sciences, Rafsanjan, Iran; 5 Department of Fundamental Nursing, Geriatric Care Research Center, Faculty of Nursing and Midwifery, Rafsanjan University of Medical Sciences, Rafsanjan, Iran; 6 Department of Medical Surgical Nursing, Jovein School of Nursing, Sabzevar University of Medical Sciences, Sabzevar, Iran

**Keywords:** Pain, Cold compress, Transcutaneous electrical nerve stimulation, Chest tube, Coronary artery bypass grafting

## Abstract

**Background and aim:**

Chest tube removal (CTR) can cause severe acute pain which is usually described by patients as a painful experience. This study compared the effects of cold compress, transcutaneous electrical nerve stimulation (TENS), and combined cold compress-TENS on CTR-associated pain among patients with coronary artery bypass grafting (CABG).

**Methods:**

This randomized controlled trial was conducted in 2018–2019 using a double-blind four-group design. Participants were 120 patients with CABG selected from Shafa hospital, Kerman, Iran, and randomly allocated to a cold compress, a TENS, a combined cold compress-TENS, and a placebo group (compress with room temperature) and TENS with an off TENS device. Each participant received the intervention for 15 min immediately before CTR. CTR-associated pain was assessed before, during, immediately after, and 15 min after CTR. Data were analyzed using the SPSS program (v. 22.0) at a significance level of less than 0.05.

**Results:**

The data of 29 participants in the placebo group, 26 in the TENS group, 30 in the cold compress group, and 26 in the combined cold compress-TENS group was gathered. Baseline demographic and clinical characteristics and pain intensity scores of participants had no statistically significant differences among all four groups (*P* > 0.05). The mean score of pain intensity in all groups was at its highest level during CTR and gradually decreased afterwards, but this pain intensity reduction in the compress-TENS group was significantly greater than other groups (*P* < 0.001).

**Conclusion:**

Combined cold compress-TENS is more effective than separate cold compress and TENS in reducing CTR-associated pain among patients with CABG. Therefore, non-pharmacological methods such as combined cold compress-TENS are recommended for managing CTR-associated pain.

## Introduction

Cardiovascular disease is one of the major health challenges [1] and a leading cause of disability and death worldwide [2]. Estimates show that cardiovascular disease is the leading cause of 45% of all deaths [3]. In 2017, cardiovascular disease caused around 17.8% deaths in the world [4, 5]. The prevalence and the mortality rates of cardiovascular disease in Iran are 23.3% and 46%, respectively [6].

Cardiac surgery is a common treatment for cardiovascular disease [7, 8]. Each day, thousands of people undergo cardiac surgery in the United States [9]. In Iran, 35–50 thousand cardiac surgeries are performed each year [6]. In cardiac surgery, a chest tube is used to remove secretions and improve cardiac function which is usually removed during the first 24–48 h after surgery [10]. Chest tube removal (CTR) may be associated with a severe transient pain which is among the most painful experiences of patients with cardiac surgery [11]. Due to the abundance of sensory fibers in the pleura, the pain of CTR may be associated with burning, tension, and pressure sensations and may cause fear and anxiety [12, 13].

Ineffective pain management among patients with cardiac surgery can increase the risk of postoperative respiratory complications such as reduced respiratory muscle strength, reduced lung volume and capacity, reduced effectiveness of coughing, and increased risk of respiratory infections. These complications negatively affect postoperative recovery and are among the major causes of postoperative death [10]. Contrarily, effective postoperative pain management facilitates recovery, reduces patient discomfort, and shortens the length of hospital stay [14, 15].

There are many different pharmacological and non-pharmacological pain management methods. Pharmacological methods (such as opioids and non-steroidal anti-inflammatory drugs) are usually associated with different side effects such as dyspnea, nausea, and gastrointestinal bleeding. Therefore, non-pharmacological methods are usually preferred for pain management [16–18]. These methods maintain patient autonomy, are easy to use, and usually have limited side effects [19]. Non-pharmacological methods for pain management include acupressure, massage therapy, muscle relaxation, cold therapy, and transcutaneous electrical nerve stimulation (TENS).

Cold compress is a common non-pharmacological method for managing CTR-associated pain. It is among the oldest, easiest, and safest pain management methods [10]. Cold compress reduces pain through reducing the velocity of pain signal transmission to the central nervous system [20, 21], reducing local blood flow, reducing cell metabolism, and reducing tissue injury [22]. In fact, cold reduces inflammation, constricts deep vessels, reduces the release and transmission of chemical pain mediators, and thereby, raises pain threshold and reduces pain [23].

TENS is another non-pharmacological method for pain management [24] which has been used since 1974 as a safe pain management method [25]. In this method, mild electrical current is applied to the skin which is supposed to stimulate efferent fibers, inhibitory neurons in the dorsal horn, the descending inhibitory system, and the release of endorphins, and thereby, prevents pain signal transmission to the central nervous system. Electrical stimulation can also increase local blood flow and thereby, indirectly reduce pain through facilitating the healing of injuries and the relaxation of muscle spasms [26, 27]. TENS is a safe non-invasive method for pain management which can easily be used by nurses. Unlike other non-pharmacological pain management methods, the use of TENS requires neither specialized training nor intense patient preparation [28].

Several studies reported the effectiveness of cold compress in reducing CTR-associated pain [13, 14, 29, 30], while several other studies reported its insignificant effects [21, 31, 32]. On the other hand, a study showed that TENS was ineffective in reducing CTR-associated pain [33], while another study reported that TENS significantly reduced postoperative pain with less effectiveness than parasternal block [34]. The contradictory results of previous studies into the effects of cold compress and TENS and the lack of comparative studies in this area highlighted the necessity of conducting more studies for producing more conclusive evidence. Therefore, the present study aimed to evaluate and compare the effects of cold compress, TENS, and combined cold compress-TENS on CTR-associated pain among patients with coronary artery bypass grafting (CABG).

## Methods

This randomized controlled trial was conducted in 2018–2019 using a double-blind four-group design. Participants were 120 hospitalized adult patients with CABG recruited from the cardiac surgery intensive care unit of Shafa hospital, Kerman, Iran. They were selected based on the following selection criteria: an age of 18–75 years, Iranian nationality, full consciousness, hemodynamic stability, sensitivity to cold, no use of tranquilizers or analgesics 1 h before CTR, no cigarette smoking or drug abuse, and no self-report history of Raynaud’s disease, sensory disorders (such as audiovisual impairments), and mental disorders, no previous experience of using TENS before the study. Exclusion criteria were the need for analgesics during the intervention or the development of cardiac dysrhythmia or vasovagal response during CTR. All patients were selected in the morning shift and underwent CABG with an identical surgical procedure performed by an identical surgeon. The size of chest tube for all of them was 14–16 French. All participants and the person who performed pain assessment were blind to the group allocation, but we explained the purpose of the study to the participants and after finishing the procedure they were given an educational pamphlet about TENS and cold therapy.

Sample size was calculated to be thirty using the results of a former study [35] and with a type I error of 5% and a type II error of 10%. Block randomization was used to allocate participants to a cold compress, a TENS, a combined compress-TENS, and a placebo group. Block randomization ensures the equal number of participants in each group. In control group, we did all the TENS procedure steps but with a device which was turned off, and a compress pad without being cold. An expert coronary ICU nurse was in charge of the intervention and the measurement of results [6].

### Instruments

Data collection instruments were a demographic questionnaire, a clinical data sheet, and a visual analogue scale. The items of the demographic questionnaire were related to age, gender, educational level, body mass index, occupation, pain tolerance, history of chronic disease, history of cigarette smoking, and family history of coronary artery bypass graft surgery. The clinical data sheet included items on chest tube characteristics, namely chest tube size, duration of chest tube use, and chest pain. The visual analogue scale for pain assessment was scored from zero (“No pain”) to 10 (“Highest possible pain”). This scale has been used in previous studies for pain assessment among patients with cardiac surgery [12, 29] and is the most widely used pain assessment instrument in the world. The most important characteristic of the scale is its simplicity [36]. The validity and reliability of this scale were confirmed in different studies [37, 38].

### Intervention

Study intervention for all participants in all groups was implemented 15 min before CTR. In the cold compress group, the skin surrounding the chest tube insertion site was cleansed using alcohol and then, a cold compress with a temperature of – 5 °C was applied to the site for 15 min so that it covered an area of 10-cm diameter around the chest tube insertion site. A sterile gauze was placed between the skin and the cold compress in order to prevent the direct contact of the compress with the skin. In the TENS group, a TENS device named stimulator 615 k (made by Novin company, Isfahan, Iran) with two pads sized four by 6 cm was used. TENS electrodes were placed on the skin next to the chest tube insertion site and TENS was started at a frequency of 80–100 Hz, the mode of the device was on normal mode and the intensity of the device was set to 6 mA [39] and a current intensity according to the participant’s tolerance as determined by a physical therapist. In the combined compress-TENS group, both cold compress and TENS were applied for 15 min. In the placebo group, TENS electrodes and compress were simultaneously applied for 15 min while the TENS device was off and the compress had a room temperature. Chest tube in all groups was removed 15 min after the intervention onset using an identical technique and under the supervision of an experienced hospital nurse. The intensity of CTR-associated pain was assessed in all groups at four time points, namely before, during, immediately after, and 15 min after CTR. All participants were provided with explanations about cold compress, TENS, and pain scale answering.

### Data analysis

The data were entered into the SPSS program for Windows (v. 22.0) and were described using the measures of descriptive statistics, namely mean, standard deviation, and frequency. Groups were compared respecting participants’ demographic and clinical characteristics using the Chi-square, Fisher’s exact, and Kruskal–Wallis tests and the one-way analysis of variance. Moreover, the repeated measures analysis of variance was used for the within- and the between-subject comparisons of the variations of the mean score of CTR-associated pain across the four measurement time points. The level of significance was set at less than 0.05.

### Ethical considerations

This study has the approval of the Ethics Committee of Rafsanjan University of Medical Sciences, Rafsanjan, Iran (code: IR.RUMS.REC.1397.174) and was registered in the Iranian Registry of Clinical Trials (code: IRCT20131228015965N18). Permissions for conducting the study were obtained from the authorities of the above-mentioned university and provided to the authorities of the study setting. The study aim was explained for all participants and informed consent was obtained from them. They were also informed about the confidentiality of their data and the voluntariness of participation. The study intervention was implemented by an experienced nurse in the study setting. Finally, study findings were provided to the authorities of the study setting.

### Findings

**Fig. 1:**
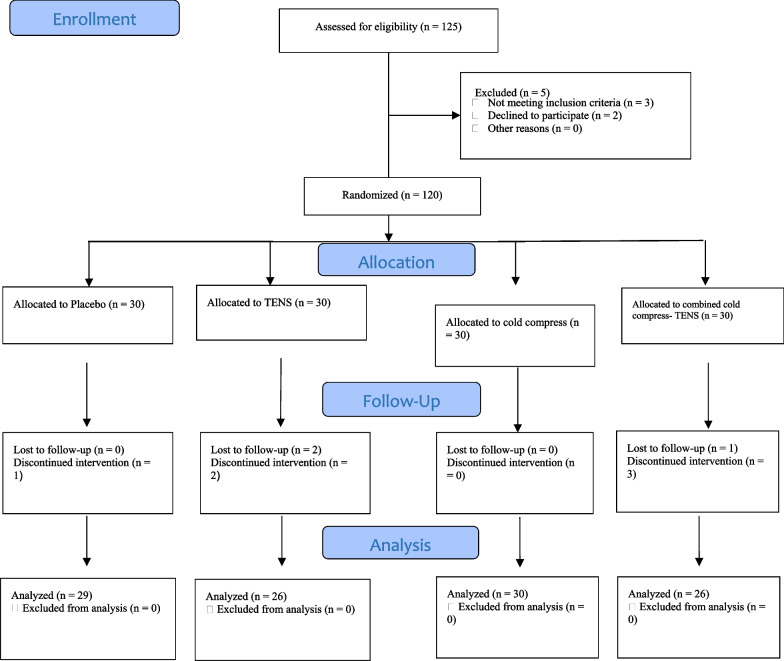
The flow diagram of the study

In total, 120 patients were recruited to the study. One participant from the placebo group, four from the TENS group, and four from the combined cold compress-TENS group were excluded from the study. Consequently, final data analysis was performed on the data obtained from 29 participants in the placebo group, 26 in the TENS group, thirty in the cold compress group, and 26 in the combined cold compress-TENS group (Fig. 1). Participants’ age mean was 57.88 ± 8.8 years and most of them were male (63%). The results of the Chi-square, Fisher’s exact, and Kruskal–Wallis tests and the one-way analysis of variance showed no statistically significant differences among groups respecting participants’ baseline demographic and clinical characteristics (*P* > 0.05) (Table 1).

**Table 1:** Comparison of the study groups respecting participants’ demographic and clinical characteristics

Characteristics		Group								
		Placebo (n = 29) N (%)		TENS (n = 26) N (%)		Cold compress (n = 30) N (%)		Combined (n = 26) N (%)	*P* value	
*Gender*										
Female		14 (3.48)		15 (7.57)		8 (7.26)		8 (8.38)	0.06^a^	
Male		15 (7.51)		11(3.42)		22 (3.73)		18 (2.69)		
*Marital status*										
Single		1 (3.4)		2 (7.77)		1 (3.3)		0 (0)	0.64^b^	
Married		28 (96.6)		24 (92.3)		29 (96.7)		26 (100)		
*Educational level*										
Illiterate		5 (17.2)		8 (30.8)		0 (0)		6 (23/1)	0.03^b^	
Below diploma		10 (5.34)		10 (5.38)		17 (56.7)		11 (42/3)		
Diploma and higher		14 (3.43)		8 (8.30)		13 (43.3)		9 (34.6)		
*Occupation*										
Unemployed		1 (4.3)		4 (4.15)		5 (7.16)		1 (8.03)	0.45^b^	
Employee		5 (17.2)		2 (7.7)		2 (6.7)		5 (19.2)		
Housewife		11 (37.9)		11 (42.3)		6 (20)		8 (8.30)		
Self-employed		8 (27.9)		7 (26.9)		13 (43.3)		10 (38.5)		
Other		(13.8) 4		2 (7.7)		4 (13.3)		2 (7.7)		
*Pain tolerance*										
Low		5 (17.9)		4 (16)		1 (3.4)		2 (8.3)	0.56^b^	
Moderate		17 (60.7)		18 (72)		21 (76.4)		17 (70.8)		
High		6 (21.4)		3 (12)		7 (24.1)		5 (20.8)		
*Family history of CABG*										
Yes		16 (57.1)		12 (48)		11 (36.7)		12 (46.2)	0.49^b^	
No		12 (42.9)		13 (52)		19 (63.3)		14 (53.8)		
*History of diabetes*										
Yes		2 (6.9)		3 (11.5)		7 (23.3)		3 (11.5)	0.35^b^	
No		27 (93.1)		23 (88.5)		23 (76.7)		23 (88.5)		
*History of hypertension*										
Yes		9 (31)		5 (19.2)		6 (20)		10 (38.5)	0.33^a^	
No		20 (69)		21 (80.8)		24 (80)		16 (61.5)		
*Atrial fibrillation*										
Yes		3 (10.3)		3 (11.5)		3 (10)		2 (7.7)	0.99^b^	
No		26 (89.7)		23 (88.5)		27 (90)		24 (92.3)		
*Respiratory disorder*										
Yes		1 (3.4)		0 (0)		0 (0)		1 (3.8)	0.60^b^	
No		28 (96.6)		26 (100)		30 (100)		25 (92)		

	Mean ± SD	Mean ± SD	Mean ± SD	Mean ± SD	*P* value
Age	55.90 ± 11.91	55.28 ± 15.10	57.03 ± 13.91	58.42 ± 14.77	0.86^c^
Weight (kg)	68.41 ± 10.34	66.92 ± 13.07	69.50 ± 11.67	69.77 ± 10.95	0.80^c^
Height (m)	167.59 ± 9.22	166.42 ± 10.26	170.43 ± 8.14	169.65 ± 7.31	0.54^d^
Body mass index	20.40 ± 2.84	20.10 ± 3.66	20.34 ± 3	20.58 ± 3.30	0.96^c^
Cigarette smoking	16.36 ± 5.04	18.57 ± 3.78	14.33 ± 5.63	16.43 ± 4.97	0.32^d^
The duration of chest tube use	3.66 ± 0.90	3.73 ± 0.87	3.83 ± 1.18	3.92 ± 0.89	0.76^c^

^a^The results of the Chi-square test

^b^The results of the Fisher’s exact test

^c^The results of the one-way analysis of variance

^d^The results of the Kruskal–Wallis test

**Fig. 2:**
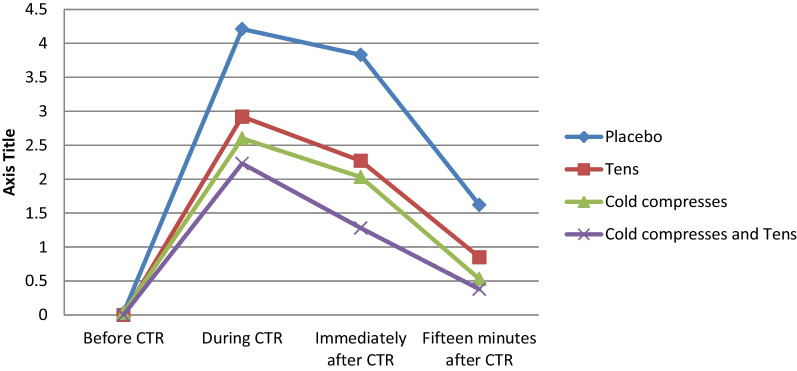
The variations of the mean score of pain intensity in all four groups across the four measurement time points

The pretest mean score of pain intensity in the study groups ranged from zero to 0.03, with no significant difference among the groups (*P* = 0.62). The mean score of pain intensity in all groups was at its highest level during CTR and gradually decreased afterwards. The results of the repeated measures analysis of variance showed that the effects of group, time, and time-group interaction were statistically significant respecting the variations of the mean score of pain intensity across the four measurement time points even after controlling the confounding effects of participants’ educational level (*P* < 0.001). The mean score of pain intensity reduction in the compress-TENS group was significantly greater than other groups (*P* < 0.001; Table 2 and Fig. 2).

**Table 2:** The results of the repeated measures analysis of variance for the variations of the mean score of CTR-associated pain across the four measurement time points

Time	Group							
	Placebo		TENS		Cold compress		Combined	
	Mean	SD	Mean	SD	Mean	SD	Mean	SD
Before CTR	0.03	0.18	0.00	0.00	0.03	0.18	0.00	0.00
During CTR	4.21	1.18	2.92	0.80	2.60	0.77	2.23	0.76
Immediately after CTR	3.83	1.07	2.27	0.78	2.03	1.0	1.27	1.08
Fifteen minutes after CTR	1.62	0.77	0.85	0.67	0.53	0.73	0.38	0.57

Source	Sum of squares	*DF*	F	*P* value
Time	617.38	3	562.04	< 0.001
Group	132.91	3	37.94	< 0.001
Time-group interaction	55.37	9	16.80	< 0.001
Error	124.94	107

## Discussion

This study compared the effects of cold compress, TENS, and combined cold compress-TENS on CTR-associated pain among patients with cardiac surgery. Study findings indicated that while there was no significant difference among the groups respecting the pretest mean score of pain intensity, the variations of the mean score of pain intensity across the four measurement time points in all four groups were statistically significant. The amount of pain reduction in all three intervention groups was significantly greater than the placebo group and the combination of cold compress and TENS was more effective than both cold compress and TENS in reducing pain intensity.

Findings showed that before CTR, the intensity of CTR-associated pain was mild (range: 0–0.03). This is in line with the findings of former studies [14, 30, 32]. Compared with baseline readings, pain intensity in the present study significantly increased in all four groups of the study during CTR. Several earlier studies also showed that patients usually experience moderate to severe pain during CTR [14, 30, 31, 40–42]. CTR-associated pain is due to the stimulation of different dermal, somatic, and visceral nerve fibers [43].

We also found that pain intensity at 15 min after CTR in the compress-TENS group was significantly less than both cold compress and TENS groups. Similarly, two former studies reported that after CTR for 15 min, CTR-associated pain in the cold compress group was significantly less than the control group (Mazloom, Demir). Contrary to our findings, a study showed that cold compress had no significant effects on CTR-associated pain [32]. Another study also showed the ineffectiveness of TENS in significantly reducing CTR-associated pain among patients with thoracic surgery [33]. These contradictions may be due to the differences among studies respecting sample size, sample characteristics, and intervention duration.

Our findings also showed that although cold compress, TENS, and combined cold compress-TENS were effective in significantly reducing CTR-associated pain, the effects of combined cold compress-TENS were significantly greater than both cold compress and TENS. Combined methods are more effective than separate methods in reducing pain [14, 33]. A study showed that cold application significantly reduced CTR-associated pain but did not significantly reduce anxiety; hence, it recommended the combination of cold and standard analgesic administration for CTR-associated pain management [14]. Another study also reported the ineffectiveness of TENS in reducing CTR-associated pain and recommended the use of TENS in combination with other methods for the management of CTR-associated pain [33].

## Conclusion

This study concludes that the combination of cold compress and TENS is more effective than both cold compress and TENS in significantly reducing CTR-associated pain among patients with CABG. As independent nursing measures, cold compression and TENS are safe, inexpensive, and easy to use methods for pain management and hence, their combination is recommended for the effective management of CTR-associated pain among patients with CABG.

### Limitations and recommendations

One of the study limitations was the limited number of eligible patients in the study setting. Another limitation was the limited number of studies into the effects of cold compress and TENS on CTR-associated pain. Future studies are recommended to compare the effects of pharmacological and non-pharmacological methods on pain management among different patient populations.

## Data Availability

The data of this study will be provided to the editor of the journal if it is needed.
